# Fungal Hydrophobin Proteins Produce Self-Assembling Protein Films with Diverse Structure and Chemical Stability

**DOI:** 10.3390/nano4030827

**Published:** 2014-09-17

**Authors:** Victor C. Lo, Qin Ren, Chi L. L. Pham, Vanessa K. Morris, Ann H. Kwan, Margaret Sunde

**Affiliations:** 1 Discipline of Pharmacology, School of Medical Sciences, The University of Sydney, Sydney NSW 2006, Australia; E-Mails: v.lo@usyd.edu.au (V.C.L.); qren5387@uni.sydney.edu.au (Q.R.); chi.pham@sydney.edu.au (C.L.L.P.); vanessa.morris@tum.de (V.K.M.); 2 School of Molecular Bioscience, The University of Sydney, Sydney NSW 2006, Australia; E-Mail: ann.kwan@sydney.edu.au

**Keywords:** hydrophobin, protein, film, amphipathic, self-assembly

## Abstract

Hydrophobins are small proteins secreted by fungi and which spontaneously assemble into amphipathic layers at hydrophilic-hydrophobic interfaces. We have examined the self-assembly of the Class I hydrophobins EAS_∆15_ and DewA, the Class II hydrophobin NC2 and an engineered chimeric hydrophobin. These Class I hydrophobins form layers composed of laterally associated fibrils with an underlying amyloid structure. These two Class I hydrophobins, despite showing significant conformational differences in solution, self-assemble to form fibrillar layers with very similar structures and require a hydrophilic-hydrophobic interface to trigger self-assembly. Addition of additives that influence surface tension can be used to manipulate the fine structure of the protein films. The Class II hydrophobin NC2 forms a mesh-like protein network and the engineered chimeric hydrophobin displays two multimeric forms, depending on assembly conditions. When formed on a graphite surface, the fibrillar EAS_∆15_ layers are resistant to alcohol, acid and basic washes. In contrast, the NC2 Class II monolayers are dissociated by alcohol treatment but are relatively stable towards acid and base washes. The engineered chimeric Class I/II hydrophobin shows increased stability towards alcohol and acid and base washes. Self-assembled hydrophobin films may have extensive applications in biotechnology where biocompatible; amphipathic coatings facilitate the functionalization of nanomaterials.

## 1. Introduction

Hydrophobins are small, surface active proteins that are secreted from filamentous fungi and which self-assemble spontaneously into amphipathic layers at hydrophilic-hydrophobic interfaces [1,2]. These protein assemblies facilitate and support the fungal lifecycle at air-water and water-environmental interfaces. Hydrophobins are characterized by the presence of eight cysteine residues and the family members broadly fall into one of two main groups, the Class I and the Class II hydrophobins. Class I hydrophobins self-assemble into amphipathic layers that are composed of laterally associated and chemically robust fibrillar structures known as rodlets [3,4,5,6,7]. Class II hydrophobins self-assemble into non-fibrillar films that can be dissociated by treatment with alcohol solutions and detergents [8]. These biocompatible, self-assembling, amphipathic films show promise for use in nanotechnology applications, including coating implanted medical devices, biosensors and cell growth surfaces [9,10,11,12,13]. An understanding of the molecular basis for hydrophobin self-assembly is required in order to control the morphology and properties of these protein layers. The soluble forms of the hydrophobins share a similar structural scaffold imposed by the network of disulfide bonds, while sequence diversity between family and class members is accommodated in diverse secondary structural elements and loop regions [14]. The hydrophobins have relatively large, exposed hydrophobic patches on the protein surface and display a clustering of surface charged residues that is likely to underpin the observed high surface activity of these proteins [15,16]. We have carried out a study of the structure and stability of the layers formed by spontaneous assembly of Class I and Class II hydrophobins and an engineered chimera, which carries the rodlet-forming region of a Class I protein grafted onto the core structure of a Class II hydrophobin. The Class I hydrophobins we examined are DewA, a hydrophobin expressed in the spores of *Aspergillus nidulans*, and EAS_∆15_, an engineered variant of EAS (named for its easily wettable spore phenotype), the hydrophobin that forms a protein coating at the surface of the spores of *Neurospora crassa*. EAS_∆15_ lacks 15 residues from the long disordered region of EAS but forms rodlets with indistinguishable morphology and structural characteristics [5,17,18]. In spite of significant differences in sequence, size and molecular structure of the soluble forms of these Class I hydrophobins, DewA and EAS_∆15_ form rodlet films with very similar morphology. We have also examined self-assembly by a Class II hydrophobin from *Neurospora crassa*, NC2 [19] and an engineered chimeric hydrophobin NChi2, which carries the rodlet-forming region of EAS (this region is common to EAS and EAS_∆15_) grafted onto the core structure of NC2 [20]. Comparison of the protein layers formed by EAS_∆15_, the Class II protein NC2 and the chimeric protein NChi2 on highly oriented pyrolytic graphite (HOPG) shows that there are distinct differences in morphology and chemical resistance. While EAS_∆15_ rodlets are stable towards ethanol, 3M NaOH and 3M HCl washes and require treatment with trifluoroacetic acid (TFA) for dissociation, NC2 and NChi2 protein layers form a mesh on the HOPG surface, which is rapidly dissociated by treatment with ethanol but shows some stability towards 3M NaOH and 3M HCl. These characteristics will facilitate the use of these self-assembled amphipathic nanomaterials for biotechnological purposes.

## 2. Results and Discussion

### 2.1. EAS_∆15_ and DewA Class I Hydrophobins Form Rodlets with Similar Morphology in Spite of Differences in Protein Sequence and Solution Structure

**Figure 1:**
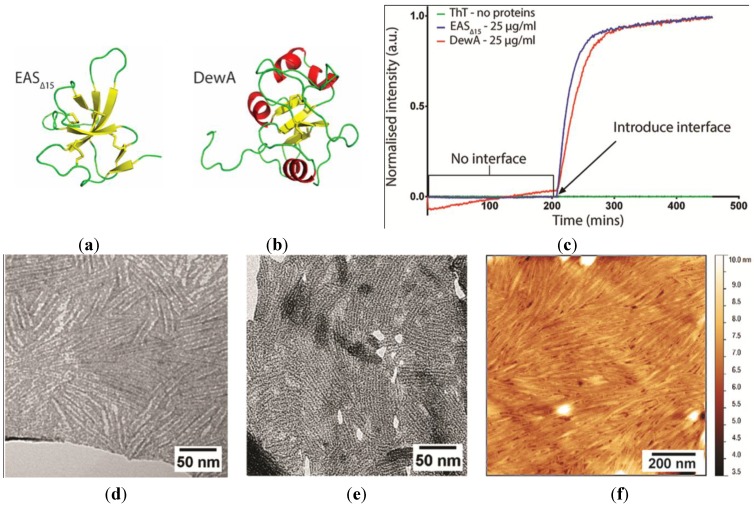
Solution structures of the soluble forms of Class I hydrophobins: (**a**) EAS_∆15_ and (**b**) DewA, determined by nuclear magnetic resonance (NMR) spectroscopy. Ribbon representations of protein molecular structure were prepared from Protein Data Bank (PDB) entries 2K6A and 2LSH using the molecular graphics program PyMol [24]. (**c**) Rodlet assembly by EAS_∆15_ and DewA assayed by Thioflavin T (ThT) fluorescence demonstrates that these Class I hydrophobins only assemble into amyloid-like rodlets after introduction of a hydrophilic-hydrophobic interface. Transmission electron microscopy (TEM) of negatively stained: (**d**) EAS_∆15_ and (**e**) DewA hydrophobin rodlet monolayers, transferred from the surface of protein droplets and imaged through the holes in a holey film. (**f**) Atomic force microscopy (AFM) of the rodlets formed when a 5 µg/mL DewA solution was allowed to dry overnight onto a highly oriented pyrolytic graphite (HOPG) surface.

We have previously shown that the solution monomeric structures of EAS_∆15_ [17] and DewA [5] display quite different secondary structure and loop elements (Figure 1a,b). Here, we have investigated the role of the hydrophilic: Hydrophobic interface in self-assembly by these two hydrophobins and compared the morphology of the protein layers formed by EAS_∆15_ and DewA. The rodlets formed by Class I hydrophobins have an amyloid substructure and therefore rodlet formation can be monitored through the increase in fluorescence that arises from binding of the amyloid-specific dye Thioflavin T (ThT) to the rodlets as they form [18,21]. We have used a ThT fluorescence-based * in vitro* system to monitor rodlet assembly by EAS_∆15_ and DewA. When the proteins are incubated with agitation in the absence of an air-solution interface (by completely filling the wells so that the plate sealing film is in contact with the surface of the solution), no ThT-positive rodlet assembly is detected. When an air gap (*i.e*. an air-solution interface) is introduced in the wells by reducing the volume of this protein solution, continued incubation with agitation results in rapid rodlet assembly (Figure 1c). This demonstrates that rodlet assembly by the Class I hydrophobins EAS_∆15_ and DewA only occurs at a hydrophilic-hydrophobic interface. These results contrast with those of Zykwinska * et al.* [4] and Longobardi and colleagues [22], who have demonstrated that the hydrophobins SC3 and Vmh2 can assemble into rodlets in solution. Transmission electron microscopy (TEM) images of EAS_∆15_ and DewA rodlet layers transferred from the surface of protein droplets onto holey films, so that the protein layers can be imaged in the absence of a supporting film, show that these two proteins form rodlets with similar dimensions and substructure (Figure 1d,e). The rodlets have a “double track” appearance; EAS_∆15_ rodlets are 6.7 ± 1.3 nm wide and DewA rodlets are 6.7 ± 0.8 nm wide, which is consistent with the model for intermolecular assembly that has been proposed for EAS rodlets [20]. When a solution of DewA is dried down onto a HOPG surface, the protein spontaneously self-assembles into rodlets (Figure 1f) with similar morphology to that seen for EAS_∆15_ [13] and other Class I hydrophobins [3,4]. Extensive structural studies are yet to reveal whether or how DewA is accommodated within a similar rodlet structure [5] but these TEM and atomic force microscopy (AFM) studies suggest that similarities in the assembly mechanism are likely.

**Figure 2:**
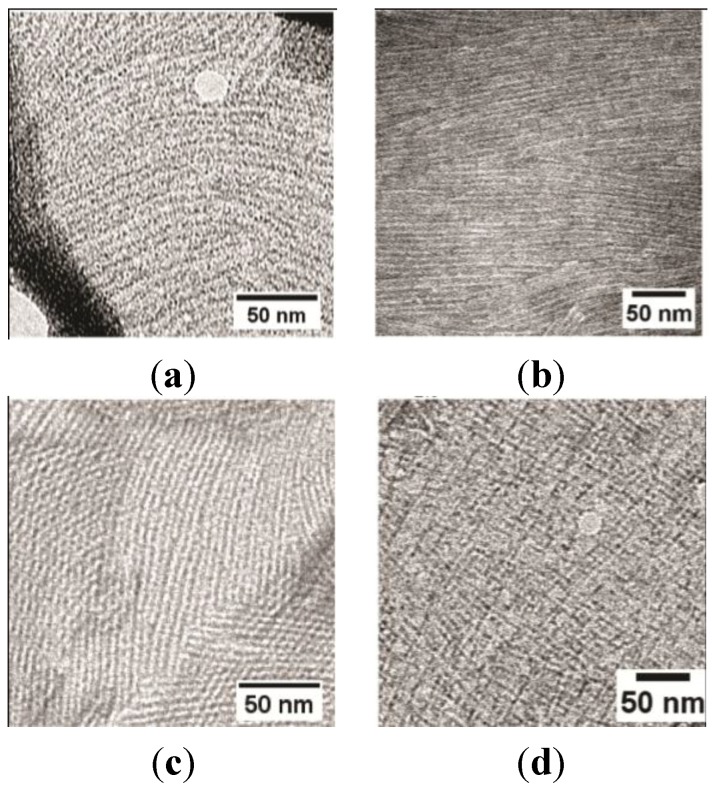
TEM image of negatively stained DewA and EAS_∆15_ hydrophobin rodlet-containing layers transferred from the surface of droplets of solutions containing: (**a**) DewA 15% (*v*/*v*) ethanol; (**b**) EAS_∆15_ 15% (*v*/*v*) ethanol; (**c**) DewA 25% (*v*/*v*) ethanol; and (**d**) EAS_∆15_ 25% (*v*/*v*) ethanol after incubation at room temperature for 20 min.

We have previously demonstrated that surface tension controls the spontaneous self-assembly of hydrophobins at the air-solution interface [23]. This work demonstrated that the addition of additives that reduce solution surface tension results in an increase in the time taken for the Class I hydrophobin rodlets to self-assemble; and in some cases, rodlet assembly was completely inhibited over the timescale of the experiment. From these results it was apparent that the Class I hydrophobins EAS_∆15_ and DewA show distinct sensitivity to the solution surface tension, indicative of the individual propensity of each of these surface-active proteins to migrate to the interface and undergo the conformation changes that result in self-assembly into the fibrillar form. We have used this sensitivity to surface tension to optimize conditions for preparation of highly ordered fibrillar films from DewA and EAS_∆15_. Inclusion of 15% ethanol in the hydrophobin solution results in the formation of more ordered single layers (Figure 2a,b) while increasing ethanol concentration to 25% tends to result in the observation of many areas displaying a cross-hatched morphology (Figure 2c,d). In TEM, this observation is consistent with two single rodlet layers lying on top of each other. There are at least two possible reasons for this morphology. The increasing ethanol in solution may alter the way in which the excess protein solution is blotted from the grid, resulting in the formation of an additional rodlet layer as the grid dries. Or, the presence of ethanol may affect the adhesion of the rodlet layer to the grid, such that as the excess protein solution is removed by blotting, patches of rodlets are mobilised and slide across on top of each other.

### 2.2. EAS_Δ15_ Forms a Single Layer Composed of Rodlets on Highly Oriented Pyrolytic Graphite

**Figure 3:**
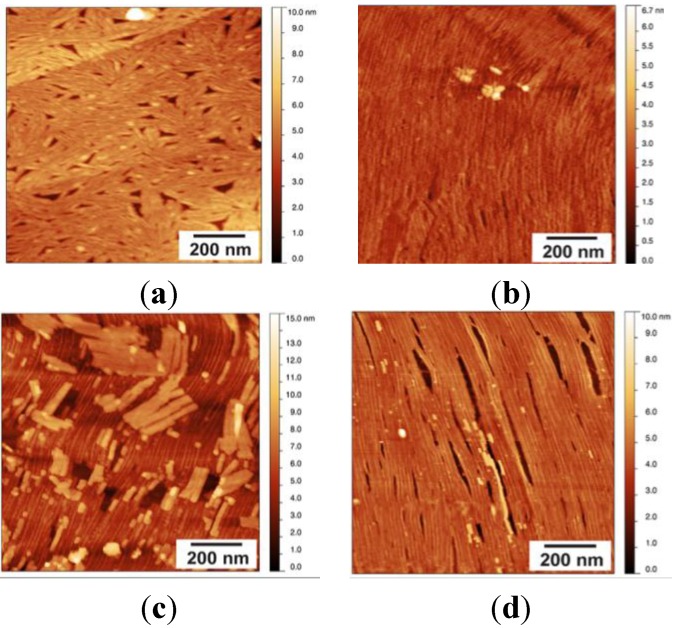
(**a**) AFM topographic scan of HOPG surface coated with a layer of EAS_∆15_ protein after a 50-µL drop of EAS_∆15_ (25 µg/mL) was incubated for 1 min and transferred onto a freshly cleaved HOPG surface; multiple protein rafts are observed; (**b**) AFM scan of HOPG surface coated with a layer of EAS_∆15_ protein after a 50 µL drop of EAS_∆15_ (5 µg/mL) was left to dry overnight onto a freshly cleaved HOPG surface. Sample was imaged directly after drying; (**c**) a 1-min wash to remove loosely bounded protein layers reveals an underlying, ordered single layer rodlet film; and (**d**) after 7 min of washing with a stream of running Milli-Q^®^ water (MQW), loose fibril layers are removed and a highly ordered layer of rodlets remains attached to the HOPG.

The rodlets formed by EAS_Δ15_ at an air-solution interface can be transferred to another hydrophobic surface, such as HOPG. Initially, EAS_Δ15_-coated surfaces were prepared for AFM imaging using a modified protocol based on the sample preparation protocol for TEM. A droplet of EAS_Δ15_ protein solution at a concentration of 25 µg/mL was incubated on PARAFILM^®^ (Bemis Company Inc. Neenah, WI, USA) for 1 min at room temperature, allowing the protein to assemble into a rodlet coating on the surface of the droplet. Assembled rodlets were transferred onto a freshly cleaved HOPG surface by contact between droplet and HOPG surface for 5 min. The surface was then briefly washed with water and air-dried before imaging. Clusters of laterally associated rodlets (“rafts”) were observed, dispersed across the HOPG surface (Figure 3a). The morphology of the rodlets observed under AFM was similar to that observed by TEM when hydrophobin layers from a droplet surface were transferred to carbon-pioloform-coated copper grids. The rodlets are hundreds of nanometres long, are distinct from the HOPG background, and are clearly visualized in both amplitude and phase images.

Class I hydrophobin rodlet formation occurs spontaneously at hydrophilic-hydrophobic interfaces, so it is also possible to assemble rodlets from solution directly onto the HOPG surface. Methods were developed which resulted in the formation of highly regular and uniform layers of EAS_Δ15_ on HOPG. The most uniform and reproducible surface was obtained by drying a dilute protein solution by evaporation directly onto the HOPG surface at room temperature, over ~16 h, followed by extensive washing with Milli-Q^®^ water (MQW, Merck Millipore, Bayswater, Australia). Initial imaging showed that when the EAS_Δ15_ coating was prepared by drying down a 50-µL drop of protein solution (at a concentration of 5 µg/mL) onto the HOPG block, a thicker protein coating, indicative of the presence of multiple protein layers, was formed on the surface (Figure 3b). The upper layers could be progressively removed by extended periods of washing, as can be seen in Figure 3c,d. When the surface was rinsed with MQW for 1 min, the upper layer(s) were disturbed and partially removed, resulting in the observation of rafts of rodlets scattered on top of the rodlet layer across the entire image (Figure 3c). Finally, when the surface was washed for 7 min with a stream of running MQW, a very uniform single layer of protein rodlets could be obtained (Figure 3d). Almost all of the examined surface area is covered with rodlets, which are microns long, unbranched and all oriented in one direction. The rodlets formed under these conditions by EAS_∆15_ are longer and display a more regular lateral packing than those reported in the literature from other Class I hydrophobins but have similar width and height [4,6,25].

#### Stability of the EAS_Δ15_ Rodlet Layer

There have been many reports that indicate Class I hydrophobin rodlets are extremely robust towards chemical treatments and can only be dissociated to a monomeric form by treatment with concentrated TFA or formic acid [20,26]. Indeed, this has been taken as one of the defining features of Class I hydrophobins, since it contrasts with the films formed by Class II hydrophobins, which can be disrupted by treatment with alcohol or detergent solutions [26]. However, most of these characterizations have been made on the basis of treatment of pre-assembled hydrophobin rodlets in solution with a range of chemicals followed by analysis of the solution using sodium dodecyl sulphate-polyacrylamide gel electrophoresis (SDS-PAGE). Hydrophobin rodlets are not dissociated or solubilized by treatment with reducing sample buffer and therefore do not electrophorese through a SDS-PAGE gel. However, the monomeric forms of the hydrophobins can be mobilized and visualized by SDS-PAGE [27,28]. In order to directly examine the chemical stability of the rodlet layers, AFM was used to visualize the morphology of the rodlets upon treatment with 60% or 100% (*v*/*v*) ethanol, 3M NaOH, 3M HCl, and 100% TFA. These test conditions were chosen for investigation as they have been used to probe the stability of hydrophobin assemblies in other studies [29,30]. The EAS_Δ15_ coatings were prepared as described above, by drying a dilute protein solution onto HOPG, followed by washing for 6 min with running MQW. The surfaces were then submerged in the test solution for 5 min before further washing with MQW to remove any loosely bound protein.

**Figure 4:**
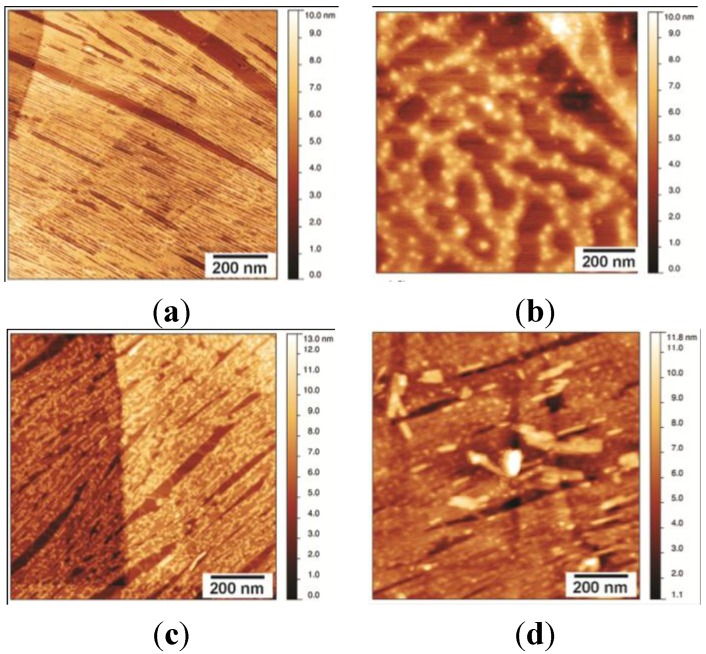
AFM scans of EAS_∆15_ rodlets on HOPG treated for 5 min with different solvents, followed by a 2-min water wash: (**a**) exposure to up to 100% ethanol does not affect the structure of the EAS_∆15_ rodlet layer and effectively cleans the surface of any loosely bound protein; (**b**) EAS_∆15_ fibrillar film is disrupted by treatment with 100% trifluoroacetic acid (TFA); fibrillar rodlet structure is stable towards treatment with: (**c**) 3M NaOH and (**d**) 3M HCl, but residual impurities remain attached to the fibrils and are not easily removed by washing.

Treating the EAS_Δ15_ rodlet films on HOPG with 60% or 100% (*v*/*v*) ethanol efficiently washed off any loosely bound protein but did not disturb the layer in direct contact with HOPG. The process resulted in increased uniformity of height across the HOPG-bound rodlet layer (Figure 4a). Treatment with 100% TFA completely disrupted the fibrillar morphology of the rodlet layer and reduced adherence of the protein to the graphite surface (Figure 4b). The loss of the fibrillar structure was accompanied by the aggregation of the protein into large, globular structures. Treatment of the rodlet layer with 3M NaOH or 3M HCl did not generally dissociate the rodlet layer from the HOPG or disturb the fibrillar structure as seen by the presence of the underlying fibrillar structure in the images (Figure 4c,d). However, residual salts or impurities remained attached to the layers that were not readily removed by washing with water.

### 2.3. NC2 Self-Assembles into a Protein Mesh

**Figure 5:**
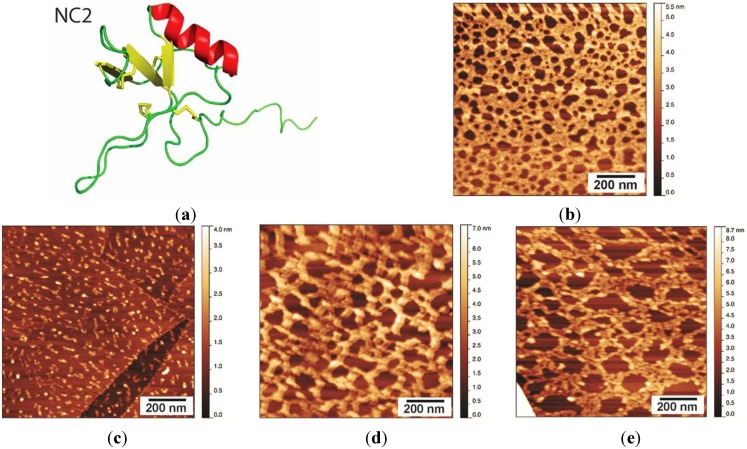
(**a**) Ribbon representation of the solution structure of NC2, prepared from PDB Entry 4AOG using PyMol [24]; (**b**) NC2 layer washed with MQW for 5 min, displaying a protein network with pores of 20–30 nm and a layer height of 1.5–2 nm; (**c**) NC2 layer after treatment with 60% ethanol for 5 min; (**d**) NC2 layer after treatment with 3 M NaOH; and (**e**) NC2 layer after treatment with 3 M HCl.

We have previously reported the first study of the structure and self-assembly of the Class II hydrophobin NC2 from *Neurospora crassa* (Figure 5a; [19]). This protein shows a high level of sequence homology to Class II hydrophobins from other fungi and has the pattern of cysteine distribution along the polypeptide chain that is characteristic of other Class II hydrophobins. The protein has been shown to be highly surface active, consistent with a large hydrophobic area on one face of the protein and a clustering of charged residues on the opposite face of the protein [19]. When NC2 is prepared on HOPG under the same conditions as described above for EAS_Δ15_, the protein forms an interconnected mesh with pores of diameter 20–30 nm and a height of 1.5–2 nm (Figure 5b). This morphology resembles that formed by the Class II hydrophobin HFBI when the films are prepared by compression with a Langmuir trough, which compresses the hydrophobin film laterally [8]. This results in a regular and uniform protein lattice with repeating hexagonal units of diameter 20–30 nm^2^.

#### Stability of the NC2 Layer

Other Class II hydrophobins have shown promise as biocompatible coatings in many biotechnology applications, including modifying electrodes for use in harsh chemical environments and as solubilising agents for poorly water-soluble drugs [11,31]. We have therefore characterized the structure and stability of the coatings formed by the Class II hydrophobin NC2, towards a range of solution conditions.

NC2 coatings were prepared as described in Section 2.2 and then incubated with 60% (*v*/*v*) ethanol, 3M NaOH, or 3M HCl at 25 °C for 5 min. After treatment, the surfaces were washed extensively with MQW to remove any solubilized and loosely bound protein, and then dried in air. As reported for other Class II hydrophobin protein coatings, the NC2 film was dissolved upon incubation with 60% (*v*/*v*) ethanol (Figure 5c). After treatment with ethanol, only small and scattered protein aggregates could be observed on the HOPG surface. The treatment with 3M NaOH and 3M HCl had a less drastic effect on NC2 assemblies, as more protein remained bound to the surface after treatment (Figure 5d,e). The morphology of the layers was disturbed by the extremely acidic and basic conditions, with larger uncoated HOPG regions visible after the treatments; however, the remaining protein layer displayed some level of organization and was not completely amorphous. While extremely acidic and basic conditions would be expected to affect salt bridges and hydrogen bonds, treatment with ethanol is likely to disrupt hydrophobic interactions. Therefore, these observations suggest that the NC2 monomers mainly interact with each other and form films via hydrophobic interactions. In addition, the NC2 layer binds to the hydrophobic HOPG surface via hydrophobic interactions, explaining why such a dramatic effect was observed upon ethanol treatment. By comparison, neither the lateral assembly within Class I rodlet films, nor the interface between protein layer and hydrophobic surface, is disrupted by ethanol treatment. This may be due to more extensive and stronger contacts between the constituent hydrophobins within the rodlets and also between the protein layer and the hydrophobic surface.

### 2.4. Self-Assembly by the Chimeric Hydrophobin NChi2 on Highly Oriented Pyrolytic Graphite

**Figure 6:**
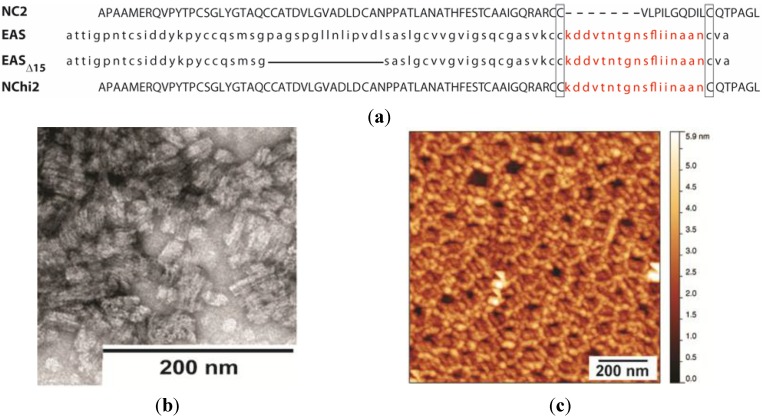
(**a**) Sequence alignment illustrating the construction of the chimeric hydrophobin NChi2 from the proteins NC2 and EAS (named for its easily wettable spore phenotype). The Cys7–Cys8 region of NC2 is shorter than the corresponding region in EAS and EAS_∆15_, this difference is indicated with a dotted line. The 15-residue deletion from EAS, to generate EAS_∆15_, is indicated by a solid line. The conservation of the amyloidogenic region between EAS, EAS_∆15_ and NChi2 is indicated in red; (**b**) TEM image of negatively stained NChi2 rodlets formed under conditions of low pH (=2.5) and elevated temperature (45 °C) with extended shaking. (**c**) AFM image of NChi2 dried down from pH = 2.5 at 45 °C and then washed with MQW for 5 min.

As part of our analysis of the sequence-structure relationships within the hydrophobin protein family, we prepared a chimeric hydrophobin with the main part of the protein composed of the NC2 protein and the sequence between the Cys7 and Cys8 replaced by the corresponding sequence from EAS (Figure 6a) [20]. This protein is able to self-assemble into short fibrillar structures, but only when incubated for extended periods at low pH and high temperature with shaking (Figure 6b), unlike EAS and EAS_Δ15_, which readily assemble into rodlets under most native conditions. This work demonstrated that the region between Cys7 and Cys8 can drive protein self-assembly into rodlet-like structures when grafted onto the core structure of the Class II hydrophobin NC2. For practical reasons, it is not possible to replicate these solution conditions when preparing protein layers on HOPG. However, when the protein is dried down from a solution at pH = 2.5 and incubated at 45 °C, NChi2 self-assembles into a mesh-like protein layer that is more similar to the protein film produced by NC2 than to the rodlet layer produced by Class I hydrophobins (Figure 6c). The thickness of the layer is ~2 nm, which is consistent with the thickness of a single hydrophobin layer. However, in some areas of the same sample a protein layer of ~4 nm was observed, possibly a bilayer of NChi2 that is stable enough to resist extensive washing by water.

#### Stability of the NChi2 Layer

**Figure 7:**
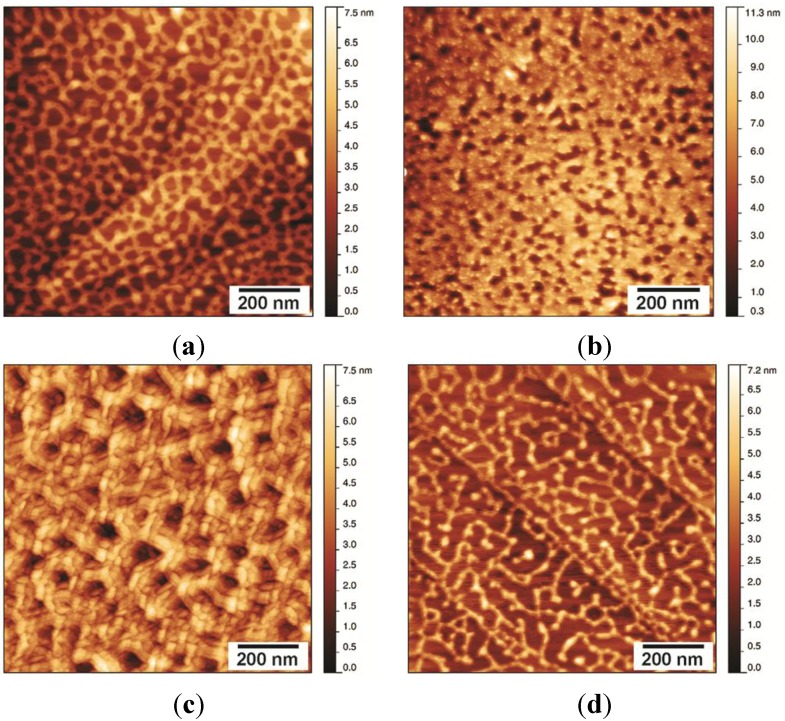
Morphology and stability of NChi2 layers formed from protein at under native conditions: (**a**) NChi2 coating washed with MQW has Class II morphology; (**b**) treatment with 60% ethanol does not disrupt the layer; (**c**) the protein network attached to HOPG is stable towards 3M NaOH; and (**d**) treatment with 3M HCl disrupts interactions between protein molecules, loosening the mesh structure.

Drying a solution of NChi2 in water onto HOPG under native conditions resulted in the formation of layers with a Class II-like morphology (Figure 7a). Treating NChi2 films formed in this way with 60% (*v*/*v*) ethanol disrupted the morphology of the NChi2 coating to a certain extent, but did not fully solubilise the layer (Figure 7b). Treatment with 3M NaOH did not appear to significantly disturb the mesh morphology (Figure 7c). A much larger change in the morphology of the layer was observed when the sample was incubated with 3M HCl (Figure 7d). In some areas of the acid-incubated NChi2 layer, the protein network was significantly disturbed and became substantially thinner, with fewer inter-protein connections visible.

Production of rodlets by NChi2 requires extended periods of incubation at elevated temperatures, conditions that are difficult to achieve during preparation of the surface from a small volume protein droplet. It is therefore likely that the NChi2 layers formed on HOPG do not contain the intermolecular β-sheet structures that are found in the Class I rodlets, and which are likely to contribute to the stability of the rodlets.

## 3. Experimental Section

### 3.1. Hydrophobin Production

All hydrophobins were produced recombinantly in *E. coli* BL21 (DE3) as His_6_-ubiquitin-hydrophobin fusion proteins, then refolded *in vitro* to a functional structure with four intact disulfides and cleaved from the His_6_-ubiquitin fusion tag before final purification by reverse-phase HPLC and confirmation of correct folding by ^1^H one-dimensional nuclear magnetic resonance (NMR) spectroscopy. Expression, purification and refolding was carried out as described for EAS_Δ15_ [17] DewA [5], NC2 [19] and NChi2 [20]. Protein concentration was determined by Pierce BCA bicinchoninic acid assay (Thermo Fisher Scientific, Scoresby, Australia) or using the appropriate extinction coefficient where the protein contained aromatic residues. Samples for TEM and AFM were prepared in filtered MQW at the desired concentration.

### 3.2. Thioflavin T Assay

ThT assays were performed with 25 µg/mL hydrophobin and 40 µM ThT in 50 mM Tris. HCl, pH = 8.0 (for EAS_∆15_) or 50 mM sodium acetate, pH 5.0 (for DewA). To investigate the role of the interface, microplate wells were filled to overflowing with hydrophobin/ThT solution (>400 µL) and then wells were sealed with sealing film. The complete removal of air from the wells was achieved by adding additional solution into the well by injection with a needle through the film. The plate was then resealed. The assay was performed at room temperature in a BMG LABTECH POLARstar Omega Microplate Reader (Mornington, Australia) with 440–10 nm excitation filter and 480–10 nm filter for detection of emission. Double orbital mixing mode at 700 rpm was continued between every data collection point. After 200 min of incubation, 100 µL of the protein solution was removed by aspiration to introduce an air-solution interface and agitation was continued.

### 3.3. Transmission Electron Microscopy

For TEM a drop of hydrophobin solution (20 µL of 50 µg/mL of DewA or 20 µL of 100 µg/mL of EAS_Δ15_) was incubated in air at room temperature for 20 min to allow self-assembly on the air-solution interface. A carbon and formvar-coated copper microscopy grid (200-mesh, ProSciTech, Townsville, Australia) or holey carbon grid (200-mesh, ProSciTech) was floated on top of the protein drop for 1 min, then excess solution was removed by touching the edge of the grid to filter paper. The grid was then washed by touching to three drops of MQW, with removal of water by wicking with filter paper between drops, and then stained with 2% uranyl acetate solution for 10 min. Excess stain was wicked off, and the grids were air dried. Grids were analyzed with a Philips CM 120 Biofilter TEM (Philips, Eindhoven, The Netherlands) operating at 120 kV and images were collected with the Gatan Imaging Filter System and Camera (Warrendale, PA, USA).

### 3.4. Atomic Force Microscopy

For AFM analysis of hydrophobin layers formed on the surface of droplets, a droplet of EAS_Δ15_ protein solution at a concentration of 25 µg/mL was incubated on Parafilm^®^ for 20 min at room temperature then transferred onto a freshly cleaved HOPG surface by contact between droplet and HOPG surface for 5 min. The surface was then briefly washed with MQW and air-dried before imaging. For preparation of protein layers *in situ* on HOPG, lyophilised hydrophobin protein was dissolved in filtered MQW to a final concentration of 5 µg/mL, and then a 50-µL droplet was placed on the surface of a block of freshly cleaved HOPG (Holgate Scientific, Terrigal, Australia), which was obtained by peeling off the top layer of the HOPG block by adhesion to sticky tape. The protein solution was incubated in air at room temperature with protection from dust, and allowed to dry completely. Coated surface was then washed with a stream of running MQW for 6 min (EAS_Δ15_ and NChi2) and 2 min (NC2), and then air-dried. To prepare NChi2 samples at pH = 2.5 and 45 °C, lyophilized protein was dissolved in 50 mM glycine (pH = 2.5), and a 50-µL drop of the sample was dried on freshly cleaved HOPG surface at 45 °C, and the coated area was washed with MQW for 6 min, and then air-dried. Any variations in sample preparation are indicated in the legends of relevant figures.

The morphology of the prepared HOPG surface was characterized at ambient atmosphere, using a Multimode Nanoscope^®^ III AFM (Veeco, Santa Barbara, CA, USA) operated in tapping mode. The AFM probes used were silicon-scanning probes with a tip radius <10 nm, operated with a force constant of 40 N/m and resonant frequency of 300 kHz (Tap300AI-G, BudgetSensors™, Innovative Solutions Bulgaria Ltd., Sofia, Bulgaria). Analysis of AFM images was performed with the Gwyddion Software (Czech Metrology Institute, Brno, Czech Republic [32]).

## 4. Conclusions

**Figure 8:**
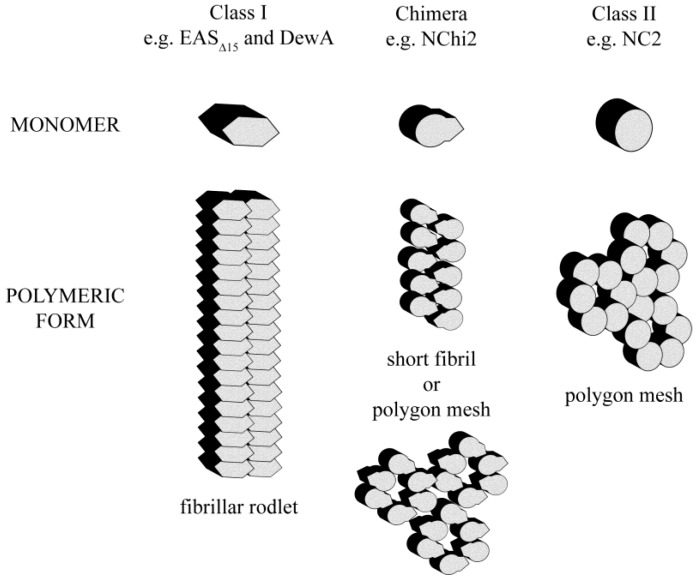
Schematic representation of the monomeric and self-assembled forms of Class I, Class II and chimeric hydrophobins. Grafting of the fibril-forming segment from a Class I hydrophobin onto a Class II hydrophobin can confer additional self-assembly character, as evidenced by the chimera NChi2 which is able to form both a Class II-like polygonal protein network or Class I-like fibrillar rodlet structures depending on assembly conditions.

Despite a similar disulfide-linked structural scaffold, Class I, Class II and chimeric hydrophobins self-assemble at interfaces into single protein layers with distinct morphologies (Figure 8).

We have demonstrated that recombinant preparations of two Class I hydrophobin proteins readily self-assemble at hydrophilic-hydrophobic interfaces into layers with a very similar fibrillar structure despite the large differences in protein sequence and solution structure. Solution conditions can be tuned to generate highly ordered and uniform protein films. These Class I films adhere tightly to hydrophobic surfaces such as graphite and are stable to alcohol washes and to 3 M NaOH and 3 M HCl washes but can be dissociated by treatment with concentrated TFA. In contrast, the Class II hydrophobin NC2 readily assembles onto a hydrophobic surface to form an extended protein network that lacks fibrillar morphology. This protein film is not stable when treated with 60% ethanol but displays some resistance towards acid or base washes. We have demonstrated that the chimeric hydrophobin NChi2 can form either a fibrillar rodlet structure or a polygonal protein network, depending on incubation conditions. The NChi2 layers deposited onto HOPG under native conditions appear partially resistant to ethanol and base washes but are more disturbed by acid wash. The morphology of the NChi2 assemblies formed under conditions of low pH and elevated temperature is more similar to the morphology of the structures formed by NC2 than to that of the rodlets formed by EAS_Δ15_ protein. However, the NChi2 assemblies formed in solution at low pH and elevated temperature show enhanced fluorescence upon ThT binding, indicating the presence of an amyloid structure. Overall, therefore, the incorporation of the rodlet-forming region from EAS results in more robust intermolecular interactions between NChi2 molecules when the protein undergoes self-assembly. This investigation of Class I, Class II and chimeric hydrophobins demonstrates that the stability and fibrillar nature of the Class I hydrophobins resides in the amyloidogenic region. This is required to impart stability towards extreme pH on the assemblies. This work indicates that if the amyloidogenic region is maintained, novel functionalities can be included within hydrophobins while maintaining the ability to form robust protein coatings. In contrast, the Class II scaffold can be used to prepare protein films which are stable towards pH extremes but can be dissociated by alcohol treatment. We have previously established that the amphipathic nature of hydrophobin coatings can be used to reverse the wettability of hydrophobic surfaces and to increase the biocompatibility of nanostructures such as carbon nanotubes which may have applications in aqueous biological environments [13]. The fungal hydrophobin proteins therefore show promise as amphipathic coatings on nanomaterials, with molecular engineering being used to generate novel properties and functionalities.
